# Whole Exome Sequencing in Two Southeast Asian Families With Atypical Femur Fractures

**DOI:** 10.1002/jbm4.10659

**Published:** 2022-07-03

**Authors:** Wei Zhou, Hanh H. Nguyen, Denise M. van de Laarschot, Tet Sen Howe, Joyce S.B. Koh, Frances Milat, Jeroen G.J. van Rooij, Joost A.M. Verlouw, Bram C.J. van der Eerden, Mark Stevenson, Rajesh V. Thakker, M. Carola Zillikens, Peter R. Ebeling

**Affiliations:** ^1^ Department of Internal Medicine Erasmus Medical Center Rotterdam The Netherlands; ^2^ Department of Medicine School of Clinical Sciences, Monash University Clayton VI Australia; ^3^ Department of Endocrinology Monash Health Clayton VI Australia; ^4^ Department of Orthopaedic Surgery Singapore General Hospital Singapore Singapore; ^5^ Academic Endocrine Unit, Radcliffe Department of Medicine University of Oxford Oxford UK

**Keywords:** ATYPICAL FEMUR FRACTURE, BISPHOSPHONATES, FAMILY STUDY, GENES, OSTEOPOROSIS

## Abstract

Atypical femur fractures (AFFs) are rare complications of anti‐resorptive therapy. Devastating to the affected individual, they pose a public health concern because of reduced uptake of an effective treatment for osteoporosis due to patient concern. The risk of AFF is increased sixfold to sevenfold in patients of Asian ethnicity compared with Europeans. Genetic factors may underlie the AFF phenotype. Given the rarity of AFFs, studying familial AFF cases is valuable in providing insights into any genetic predisposition. We present two Singaporean families, one comprising a mother (1‐a) and a daughter (1‐b), and the other comprising two sisters (2‐a and 2‐b). All four cases presented with bisphosphonate‐associated AFF. Whole‐exome sequencing (WES) was performed on 1‐b, 2‐a, and 2‐b. DNA for 1‐a was not available. Variants were examined using a candidate gene approach comprising a list of genes previously associated with AFF in the literature, as well as using unbiased filtering based on dominant and/or recessive inheritance patterns. Using a candidate gene approach, rare variants shared between all three cases were not identified. A rare variant in *TMEM25*, shared by the two sisters (2‐a and 2‐b), was identified. A rare heterozygous *PLOD2* variant was present in the daughter case with AFF (1‐b), but not in the sisters. A list of potential genetic variants for AFF was identified after variant filtering and annotation analysis of the two sisters (2‐a and 2‐b), including a Gly35Arg variant in *TRAF4*, a gene required for normal skeletal development. Although the findings from this genetic analysis are inconclusive, a familial aggregation of AFFs is suggestive of a genetic component in AFF pathogenesis. We provide a comprehensive list of rare variants identified in these AFF familial cases to aid future genetic studies. © 2022 The Authors. *JBMR Plus* published by Wiley Periodicals LLC on behalf of American Society for Bone and Mineral Research.

## Introduction

Despite the use of effective and low‐cost antiresorptive amino‐terminal bisphosphonates (BPs) to reduce fragility fractures, fear of rare side effects, such as atypical femur fractures (AFFs), has reduced their uptake.^(^ ^1^ ^)^ These unusual stress fractures of the subtrochanteric and the lateral femoral diaphyseal regions occur at sites usually resilient to traumatic fracture.^(^ ^2^ ^)^ Although rare, with an estimated incidence of two and 194 per 100,000 person years for <2 years of BP use and ≥10 years of BP use, respectively.^(^ ^3^ ^)^ AFFs can be devastating to the affected individual, as well as posing a public health concern. Proposed pathophysiological mechanisms for AFFs include adverse femoral geometric parameters and unfavorable bone microarchitecture. Prolonged antiresorptive therapy may progressively alter the material properties of bone such that with increasing toughness, bones are stiffer and less resilient against mechanical loading when weightbearing—particularly at the lateral femoral diaphyseal cortex, the site of maximal loading during walking.^(^ ^4^, ^5^ ^)^ The lowered peak tolerated strain leads to microcrack development, which accumulates because healing of microdamage is impaired by antiresorptive therapy, thus precipitating femoral stress fractures such as AFFs. However, it is notable that bisphosphonate‐naïve individuals can also sustain AFFs, described in up to 22% of AFF cohorts,^(^ ^6^ ^)^ suggesting that other independent factors contribute to AFF risk.

Ethnic variation in AFF risk has also been described. Early AFF case reports arose in Asia,^(^ ^7^ ^)^ whereas Asian ethnicity comprises up to one‐half of AFF cohorts in North America.^(^ ^8^, ^9^ ^)^ Lo and colleagues^(^ ^10^ ^)^ described a hazard ratio for AFF of 6.6 in Asian compared with white BP users and Black and colleagues^(^ ^11^ ^)^ reported a hazard ratio of 4.84 in Asian users compared with white. Similarly, we identified an AFF incidence rate in Asians 3.4‐fold higher than other ethnic groups in an Australian cohort study.^(^ ^12^ ^)^ Further, ethnic variation in anatomic AFF location has also been described, being predominantly subtrochanteric in Singapore compared with diaphyseal in Sweden.^(^ ^13^ ^)^ The mechanism underlying the increased AFF risk in Asians is not known, but an unexplored possibility is that genetic factors predisposing to AFFs are more prevalent in Asian populations.

Genetic factors have been associated with AFFs, and this literature is summarized in our recent systematic reviews.^(^ ^14^, ^15^ ^)^ In support of a genetic predisposition is the rarity of AFFs, occurrence in BP‐naïve individuals, familial cases of AFFs, and case reports of AFF occurring in those with underlying monogenetic bone disorders (Table 1*A*), at times unmasking the genetic disease. It is possible that mild phenotypes of such heritable bone disease may underlie the etiology of AFFs in some patients.

**Table 1:** Genes Implicated in AFFs

(*A*) Monogenetic bone disorders in which AFFs have occurred^(^ ^14^, ^15^ ^)^	
Monogenetic disorder	Associated genes
Hypophosphatasia	*ALPL*
Osteogenesis imperfecta^a^	*COL1A1, COL1A2, CRTAP, LEPRE1, PPIB, SERPINH1, FKBP10, PLOD2, SP7*
Pycnodysostosis	*CTSK*
X‐linked hypophosphatemia	*PHEX*
Osteopetrosis^a^	*TCIRG1, CLCN7, OSTM1, PLEKHM1, SNX10, TNFSF11, TNFRSF11A, CA2*
Osteoporosis pseudoglioma syndrome	*LRP5*
X‐linked osteoporosis	*PLS3*

(*B*) Genes with low‐frequency variants identified in AFF cases	
Study	Gene list
Roca‐Ayats and colleagues^(^ ^20^ ^)^	*ALPK1, ATP6AP1, BRAT1, CD37, CHERP, COG4, CUL9, CYP1A1, EML1, ERCC6L2, FN1, GGPS1, GPR20, HEPHL1, IQCF6, KDM4C, LFNG, LRRC1, LURAP1L, MEX3D, MGA, MVD, NGEF, NKAP, NTPCR, NVL, GRMC1, POLI, SHC4, SMS, SNAPC4, SYDE2, TMEM25, TUSC2, XAB2*
Peris and colleagues^(^ ^20^ ^)^	*ALPL, CYP1A1*
Funck‐Brentano and colleagues^(^ ^21^ ^)^	*COL1A2*
Sum and colleagues^(^ ^22^ ^)^	*ALPL*
Lau and colleagues^(^ ^23^ ^)^	*CTSK*
Furukawa and colleagues^(^ ^24^ ^)^	*ENPP1, FGF23, CYP27B1, CYP3A4, SLC34A3, CYP2R1, ALPL*
Surface and colleagues^(^ ^25^ ^)^	*ATRAID*

^a^ Osteogenesis imperfecta and osteopetrosis are associated with a number of genes. Although there have been case reports of AFFs occurring in these two conditions, the specific gene involved was not provided. As such, all genes associated with the two disorders are listed in the table.

Few genetic studies, albeit with small sample sizes, have been conducted in BP‐associated AFF cohorts. In a whole‐exome sequencing (WES) study of three sisters with BP‐associated AFFs, Roca‐Ayats and colleagues^(^ ^16^, ^17^ ^)^ identified 37 rare variants in 34 genes, including two genes of interest, *GGPS1* and *CYP1A1*. *GGPS1* interacts with the mevalonate pathway, which is important in the production of cholesterols and steroidal hormones, and, critically, is targeted by the amino‐terminal BPs to reduce osteoclast action. *CYP1A1* is involved in steroid metabolism, specifically in the oxidative metabolism of estrogens. Polymorphisms in this gene have been studied for a possible association with the risk for osteoporosis and low bone mineral density (BMD) in white and Mexican postmenopausal women,^(^ ^18^, ^19^ ^)^ but results are inconsistent. Although rare variants in *CYP1A1* have been identified in two unrelated patients with AFF, rare variants in *GGPS1* have not been identified in other AFF cases outside this described family.^(^ ^17^, ^20^ ^)^ Other studies have reported rare variants in *CTSK*, *COL1A2*, *ENPP1*, *FGF23*, *CYP27B1*, *CYP3A4*, *SLC34A3*, *CYP2R1*, and *ALPL*.^(^ ^21^, ^22^, ^23^, ^24^ ^)^ In a recent study, Surface and colleagues^(^ ^25^ ^)^ identified in two out of 27 AFF patients a variant (population frequency 1.3%) in the *ATRAID* gene, which has been shown to increase cell sensitivity to BPs. Table 1*B* presents a list of genes in which low‐frequency variants were found by WES analysis and shared by three sisters with AFF in one report^(^ ^17^ ^)^ or used in candidate gene studies for AFF.^(^ ^15^, ^20^, ^21^, ^22^, ^24^, ^25^ ^)^ These genes listed in Table 1 have not been replicated or confirmed at this moment to be causal for AFF. Moreover, Garcia‐Giralt and colleagues^(^ ^26^ ^)^ recently reported 132 genes presenting a possibly damaging variant in at least two of 12 AFF patients, highlighting 12 genes involving bone metabolic functions or in AFFs that need to be studied further.

Despite a recognized increase in risk in Asians, genetic studies of Asian familial AFF cases have not yet been described. In this case report, we present two small Singaporean families in each of which two members have sustained BP‐associated AFFs. We conducted WES on DNA of three cases, performed genetic analyses using a candidate gene‐based approach as well as an unbiased variant filtering approach, and describe the potential variants of interest.

## Description of AFF Cases

**Fig. 1:**
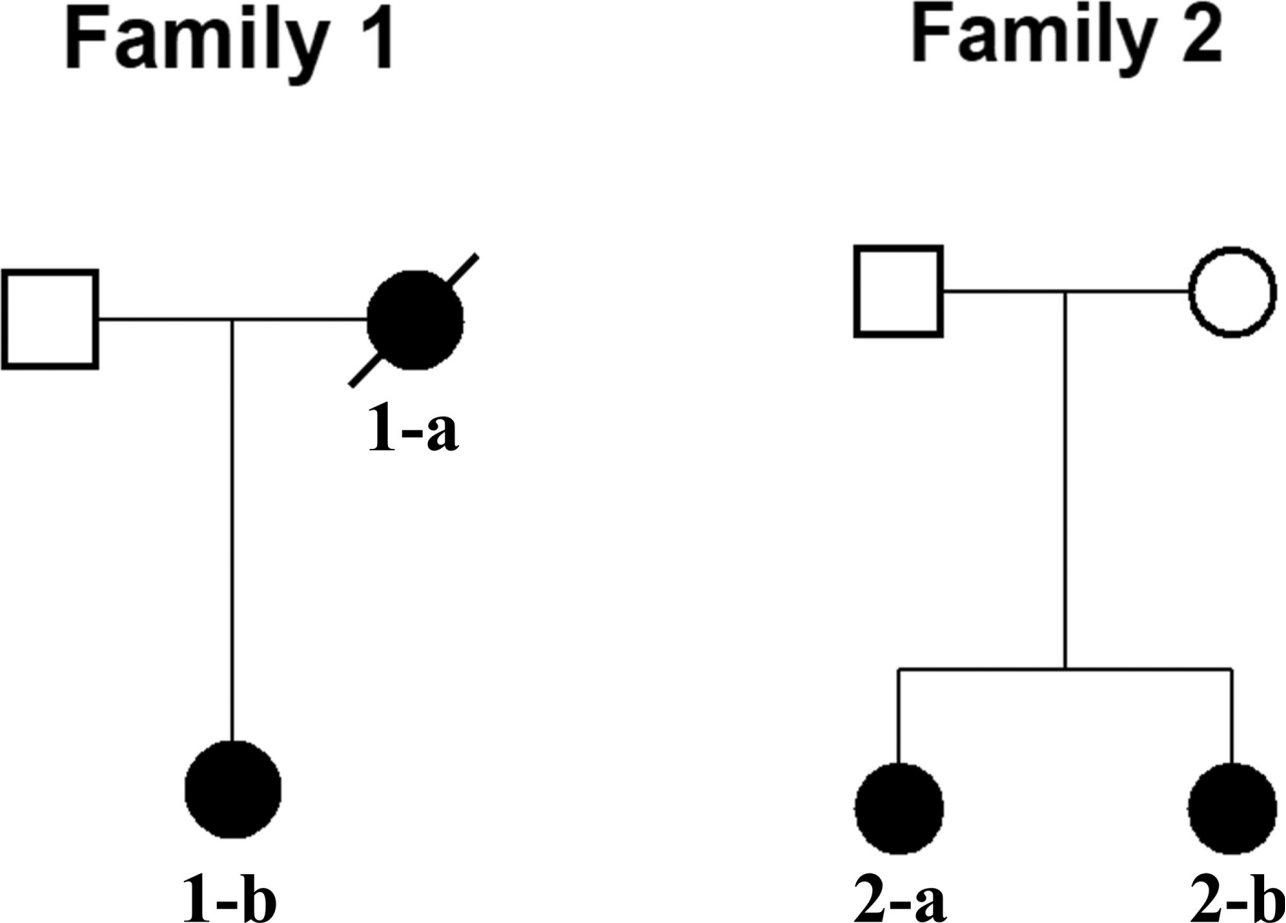
Pedigrees of two Singaporean families of Chinese origin. Black symbols represent individuals with AFF. Open symbols represent unaffected individuals.

We studied two Singaporean families of Chinese origin (Fig. 1). Family 1 comprised a mother (1‐a) and daughter (1‐b), who both sustained AFFs while on alendronate treatment. Case 1‐b is a postmenopausal woman who sustained bilateral AFFs at age 66 years following a fall from standing height requiring bilateral surgical repair. This occurred on a background of 4 years of alendronate therapy for osteopenia, without a preceding fragility fracture. Her other comorbidity included hypothyroidism, treated with levothyroxine, and being an ex‐smoker. She had no significant alcohol history.

Family 2 included two postmenopausal sisters (2‐a and 2‐b) who sustained AFFs through falls from a standing height at the age of 55 and 66 years, respectively. The sisters had been treated with alendronate for 5 and 9 years, respectively, for osteoporosis diagnosed by dual‐energy X‐ray absorptiometry (DXA) criteria, without a history of minimal trauma fractures. Neither had a significant smoking or alcohol history. The sister aged 66 years had prior menopausal hormone therapy (duration unknown), and also had received topical cortisone treatment for eczema. Genetic data was obtained from both sisters 2‐a and 2‐b.

Femoral radiographs for all four cases (1‐a, 1‐b, 2‐a, and 2‐b) were reviewed by co‐author HTS, and AFF was verified using American Society for Bone and Mineral Research (ASBMR) criteria.^(^ ^1^ ^)^ Case 1‐b, 2‐a, and 2‐b consented to take part in this study and completed structured interviews and provided genetic samples for analysis. At the time of data collection, case 1‐a (mother) had died, and her genetic data was not available. We were unable to obtain consent for genetic samples from other family members to include in the analysis.

## Subjects and Methods

### Data collection and adjudication of AFFs

The three living patients (1‐b, 2‐a, and 2‐b) consented to providing blood for DNA analysis. Clinical history was obtained via structured interviews, and AFFs were confirmed radiologically to fulfill ASBMR case definition (HTS; data not shown).^(^ ^1^ ^)^ The study was approved by Monash Health HREC (approval number 15550X).

### DNA isolation

Genomic DNA was isolated from blood samples using the Promega Reliaprep DNA isolation kit (Leiden, The Netherlands) in combination with the Tecan robot.

### WES

DNA was processed using the KAPA library preparation (Roche Diagnostics, Inc, Pleasanton, CA, USA), followed by exome capture using the Nimblegen SeqCap EZ MedExome Capture kit (Roche Nimblegen, Inc, Madison, WI, USA). Paired‐end 2 × 150‐bp sequencing was performed on the Illumina NovaSeq 6000 platform (Illumina, San Diego, CA, USA). Reads were demultiplexed and aligned to the human reference genome hg19 (UCSC) using the Burrows‐Wheeler alignment tool (BWA version 0.7.3a). After indel realignment and base quality score recalibration using the Genome Analysis ToolKit (GATK version 3.8) and masking of duplicates (Picard Tools version 2.18.4), gvcf files were generated using HaplotypeCaller (GATK v3.8) and genotyped using GenotypeGVCFs (GATK 3.8). The average WES coverage for the three samples were 62.63 (I.2), 124.09 (II.1), and 113.78 (II.2). Raw genotype data was quality controlled and filtered using the VQSR methodology of GATK. The tranche sensitivity threshold of 99.8% and 80.0% were used for filtering single‐nucleotide variants (SNVs) and insertion/deletions (INDELs), respectively. Additionally, variants with Quality of Depth (QD) <5 were removed. All detected variants were annotated based on RefSeq annotation (NCBI Reference Sequence Database) using ANNOVAR (version 2019‐10‐24). Allele frequencies from the Genome Aggregation Database (gnomAD) Exome and Genome dataset version 21120190318 were used in addition to the 1000 Genomes (version p3v5). Additionally, predictions on damaging properties of each variant were determined using Combined Annotation Dependent Depletion (CADD),^(^ ^27^ ^)^ which also includes the scores for programs such as Sorting Intolerant From Tolerant (SIFT) and Polymorphism Phenotyping (PolyPhen), and a series of conservation programs.

### Data analysis

Variants were identified by both a candidate gene‐based approach and an unbiased variant filtering and annotation of the whole exome, including inheritance pattern and suspected pathogenicity of individual variants.

In the candidate gene‐based approach, variants were identified using the list of genes implicated in AFFs (Table 1*A* and *B*). Variants were filtered based on: (i) present in the designated candidate genes (Table 1*A* and *B*); (ii) untranslated region (UTR), exonic, splicing, stopgain, stoploss, nonsynonymous or exonic indels; (iii) with a frequency <0.005 or not present in the gnomAD or 1000 genomes database; and (iv) present in either 1‐a and/or in both sisters 2‐a and 2‐b.

In the unbiased variant filtering approach, we used the two sisters (2‐a and 2‐b) in the second family to filter the variants regardless of 1‐b in the first family, because the two families may have different genetic cause for AFF. We assumed a dominant and a recessive inheritance model, respectively, and included all sequenced genes. In both models, variants were filtered based on: (i) exonic, splicing, stopgain, stoploss, nonsynonymous or exonic indels; and (ii) with a frequency <0.005 or not present in both the overpopulation and the East Asian subpopulation of the gnomAD or 1000 Genomes database. In the dominant inheritance model, variants were subsequently filtered based on heterozygosity (genotype coded 0/1) in both sisters (2‐a and 2‐b) irrespective of the variants in 1‐b. In the recessive inheritance model, variants were filtered based on homozygosity (genotype coded 1/1) or compound heterozygosity (two variants in the same gene with genotypes coded 0/1) in both sisters 2‐a and 2‐b irrespective of variants in 1‐b. The multiallelic variants were analyzed separately, where the different alternative alleles are split and annotated and interpreted manually, in line with the filtering approach indicated above.

### Gene prioritization with Kyoto Encyclopedia of Gene and Genomes pathway analysis

Because the dominant inheritance model generated a large list of genetic variants shared by the two sisters (2‐a and 2‐b), we compared the gene list to Kyoto Encyclopedia of Gene and Genomes (KEGG) pathways to identify potential candidates for AFF. Genes resulting from the filtering steps based on the dominant inheritance model were compared with genes identified in specific pathways within the KEGG database that may be of relevance to the development of AFFs. These pathways include: (i) the mevalonate pathway (M00095)^(^ ^17^ ^)^; (ii) the Terpenoid Backbone Biosynthesis pathway (map00900); (iii) KEGG osteoporosis disease pathway (H01593); and (iv) the KEGG osteoclast differentiation pathway (hsa04380).

### Analysis of Gly35Arg in TRAF4

Protein sequences of TRAF4 orthologues and paralogues were analyzed with HomoloGene (https://www.ncbi.nlm.nih.gov/homologene). The crystal structure of TRAF4 that includes the RING domain has not been reported. We therefore used the Q9BUZ4 (UniprotKB) structure generated by the AlphaFold Protein Structure Database^(^ ^28^, ^29^ ^)^ for human TRAF4. The PyMOL Molecular Graphics System (version 2.4.0, Schrodinger) was used to model the effect of the Gly35Arg mutation using the three‐dimensional AlphaFold structure archived in the Protein Data Bank at the European Bioinformatics Institute with the accession number AF‐Q9BUZ4‐F1.

### Sanger sequencing of candidate gene variants

Selected variants were confirmed with Sanger sequencing, see Fig. S1. Polymerase chain reaction (PCR) was carried out to amplify the fragments containing the variants. Primers were designed with Primer‐BLAST (https://www.ncbi.nlm.nih.gov/tools/primer-blast/). Primer sequences are listed in Table S1 and Fig. S1. Amplification was carried out at an annealing temperature of 59°C for 40 cycles. Sanger sequencing of both strands was performed at Eurofins GATC Biotech (https://www.eurofinsgenomics.eu/de/custom-dna-sequencing/gatc-services/).

## Results

WES was performed on DNA from the three female individuals of Asian origin with AFFs from the two families (1‐b, 2‐a, and 2‐b) (Fig. 1).

### Candidate gene analysis

Using the list of candidate genes (Table 1*A* and *B*), we investigated for potential interesting variants, irrespective of type of inheritance. Filtering according to the selection criteria indicated in Subjects and Methods resulted in two rare variants (Table 2), both present in a heterozygous state in either 1‐a or both sisters 2‐a and 2‐b.

**Table 2:** Analysis Flowchart of Candidate Genes With Frequency <0.005

Variable	*n*
Total number of variants^a^	49,935
All variants in candidate genes from Table 1	210
Selecting UTR, exonic nonsynonymous + splice variants (exluding intronic + exonic synonymous)	74
Variants with gnomAD WES and WGS frequency <0.005	8
Additional filtering with 1000 Genomes frequency <0.005	6
Filtering out variants only carried by one of the affected sisters 2‐a or 2‐b	2

WES = whole‐exome sequencing; WGS = whole‐genome sequencing.

^a^ Only biallelic variants included.

In the mother‐daughter AFF family, a rare variant was present in the known bone disease‐related gene *PLOD2* in individual 1‐b, but not in the sisters from the other family (2‐a and 2‐b) (Table 3). This variant (rs776654051; p.Thr419Ser) has a very low overall frequency in the gnomAD database (0.000004). Only one allele (in 250756 alleles) was found in this database and this was present in an East Asian individual. It was predicted to be tolerated by SIFT and possibly damaging by PolyPhen. The predicted CADD score of this variant was 10.8. A CADD score of 10 is low, indicating that the variant is among 10% most deleterious substitutions to the human genome, whereas a higher score suggests more confidence that the variant is damaging.

**Table 3:** Details of Rare Variants Identified Through Filtering of Candidate Genes With Frequency <0.005

Gene	Variant	Exonic function	Transcript/cDNA/protein information	dbSNP150	gnomAD WES ALL	gnomAD WES SAS	gnomAD WES EAS	SIFT score	PolyPhen2 HVAR score	CADD	GERP++	1‐b	2‐a	2‐b
PLOD2	chr3:145799628T>A	Nonsynonymous SNV	NM_182943/c.A1255T/p.Thr419Ser	rs776654051	3.99E−06	0	5.44E−05	0.88 (T)	0.515 (P)	10.78	5.53	0/1	0/0	0/0
TMEM25	chr11:118402939G>A	Nonsynonymous SNV	NM_032780/c.G145A/p.Ala49Thr	rs782188288	1.00E−04	1.00E−04	0.0013	0.55 (T)	0.209 (B)	15.48	3.26	0/0	0/1	0/1

Chromosome positions are given for build GRCh37. Both variants are not present in the gnomAD WGS and the 1000 Genome databases. SIFT score and prediction: 0–0.05 damaging (D); >0.05 tolerated (T). PolyPhen2 HVAR score and prediction: 0–0.446 benign (B); 0.446–0.908 possibly damaging (P); 0.908–1.0 probably damaging (D).

CADD = Combined Annotation Dependent Depletion tool, higher values indicate a higher chance of being damaging (max 60); GERP++ = Conservation score based on the likelihood of substitutions and the deviation thereof, higher score indicates more conservation at the site (maximum 6); HVAR = Polyphen scores trained on HumVar data meant for Mendeliandiseases (Adzhubei et al. Nat Methods 2010); WES ALL = exome data of all populations; WES EAS = exome data of East Asian population; WES SAS = exome data of South Asian population; SNV = single nucleotide variant.

In the sisters with AFF, a rare variant (rs782188288; p.Ala49Thr) in *TMEM25*, with an overall frequency of 0.00011, was shared by the two sisters 2‐a and 2‐b but was not identified in 1‐b (Table 3). The variant was predicted tolerated and benign by SIFT and PolyPhen, respectively, and had a CADD score of 15, indicating that the variant is among the 3% most deleterious substitutions to the human genome. Variants in *PLOD2* and *TMEM25* were confirmed by Sanger sequencing.

### Unbiased variant filtering

The flowcharts of the filtering approach are displayed, respectively, for the dominant inheritance model and the recessive inheritance model in Table 4. The dominant inheritance model resulted in 132 variants after filtering (Table S1). Among these, three truncating variants were present in the *EPHA10*, *SORCS1*, and *GSX2* genes, and one frameshift deletion was in the *PARP2* gene, which may result in protein truncation. Variants in the *GSX2*, *EPHA10*, *C19orf60*, *APH1B*, and *PHYHD1* genes had a CADD score >30. None of these biallelic variants was also carried by 1‐b in the first family. However, 1‐b carried one or more different variant(s) in four of the genes from the list, including *TTN*, *FAN1*, *ANKS3*, and *IL2RG* (Table S2). The recessive inheritance model resulted in the detection of a missense variant in the *MANF* gene after filtering and three pairs of compound heterozygous variants in the *SYNPO2L*, *TTN*, and *GSX2* genes (Table S3). Multiallelic variants were analyzed separately, and variants resulted from the filtering are listed in Table S4, although it did not deliver convincing candidates.

**Table 4:** Analysis Flowchart for the Dominant Inheritance Model With Frequency <0.005

Filtering step			Number of variants left
Total number of variants^a^			49,935
Selecting UTR, exonic nonsynonymous + splice variants (exluding intronic + exonic synonymous)			14,873
Variants with gnomAD WES and WGS overall population frequency <0.005			1434
Additional filtering with 1000 genomes frequency <0.005			1223
Variants with the genomAD WES and WGS and 1000 genome East Asian subpopulation freq <0.005			739
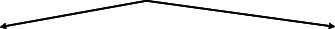			

Dominant inheritance model		Recessive inheritance model	
Filtering step	Number of variants left	Filtering step	Number of variants left
Heterozygous variants in both 2‐a and 2‐b	132	Homozygous variants in both 2‐a and 2‐b	1
		Compound heterozygous variants in both 2‐a and 2‐b	6 (in 3 genes)

^a^ Only included biallelic variants.

The current findings were compared to the 132 genes presented by Garcia‐Giralt and colleagues^(^ ^26^ ^)^ Two genes identified by the dominant model were also presented in their study, where *TTN* variants were present in eight of the 12 (67%) AFF patients and *FSIP2* variants were present in three (25%) AFF patients.

### Gene prioritization with KEGG pathway analysis

**Fig. 2:**
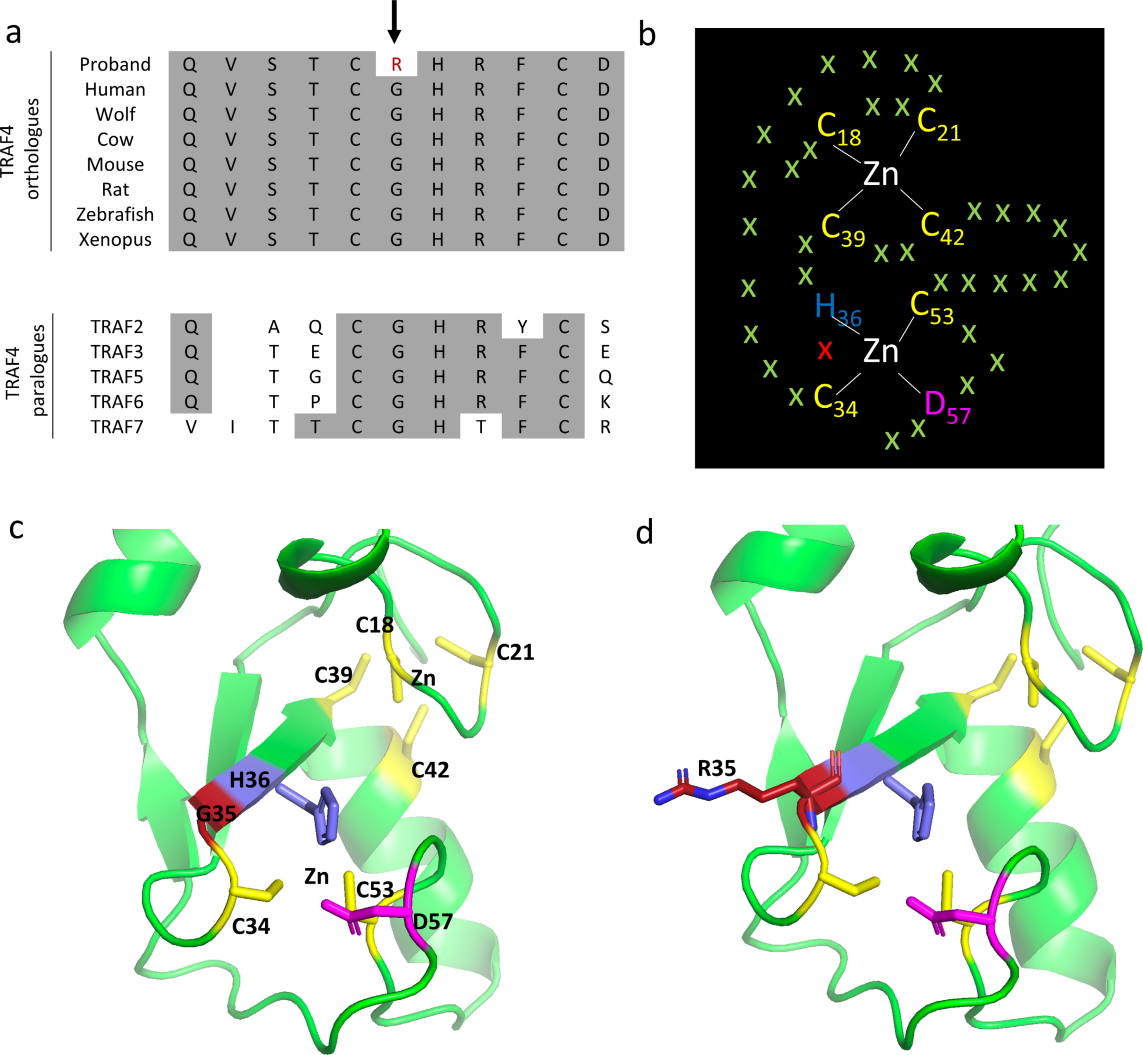
Evolutionary conservation of TRAF4 Gly35, and structural analysis of the Gly35Arg variant. (*A*) Multiple protein sequence alignment of TRAF4 revealed evolutionary conservation of Gly35 (G35) residues (indicated with an arrow) in orthologues and human paralogues. Conserved residues are shaded gray. In addition, the CGHRFC motif within the RING domain of the TRAF family of proteins is largely conserved in TRAF4 paralogues. (*B*) The RING domain of TRAF4 is predicted to contain two zinc (Zn) binding pockets involving six cysteine (C) residues (18, 21, 34, 39, 42, 53), one histidine (H) residue (36), and one aspartic acid (D) residue (57). G35 is shown as a red cross. (*C*) AlphaFold prediction of TRAF4 (AF‐Q9BUZ4‐F1) using PyMOL analysis showing the RING domain and the orientation of residues around the two zinc binding pockets. (*D*) PyMOL analysis mutating the neutrally charged small glycine residue at codon 35 to a positively charged larger arginine (R) residue, illustrating its close proximity to the zinc binding pocket, which may affect the ability of the TRAF4 RING domain to bind zinc and function as an E3 ligase.

No common genes were found between the candidate gene list resulted from the dominant inheritance model and the mevalonate pathway, the Terpenoid Backbone Biosynthesis pathway and the KEGG osteoporosis disease pathway in the KEGG database. In contrast, analysis of the KEGG osteoclast differentiation pathway revealed a potential candidate. The osteoclast differentiation pathway contains two members of the tumor necrosis factor (TNF) receptor associated factor (*TRAF*) genes, namely *TRAF2* and *TRAF6*. Although there are no variants in *TRAF2* or *TRAF6* within the AFF patients, a Gly35Arg (G35R) missense *TRAF4* variant (NM_004295.4) was present in 2‐a and 2‐b. The Gly35Arg variant was rare and only present in the East Asian population (allele frequency = 0.00005) but not present in other subpopulations in the gnomAD database. It was predicted to be deleterious and possibly damaging by SIFT and PolyPhen‐2, respectively, and had a CADD score of 17.5. A glycine residue at codon 35 was conserved in both orthologues and human paralogues, and the CGHRFC motif within the RING domain of the TRAF family of proteins is largely conserved in TRAF4 paralogues (Fig. 2*A*,*B*). The RING domain of TRAF4 was predicted to contain two zinc (Zn) binding pockets involving six cysteine (C) residues (18, 21, 34, 39, 42, and 53), one histidine (H) residue (36), and one aspartic acid (D) residue (57) (Fig. 2*B*).^(^ ^30^ ^)^ Figure 2*C* shows the RING domain and the orientation of residues around the two zinc binding pockets. Structural analysis of the Gly35Arg variant showed that the neutrally charged small glycine residue at codon 35 was mutated to a positively charged larger arginine (R) residue, which was located close to the zinc binding pocket (Fig. 2*D*). This may affect the ability of the TRAF4 RING domain to bind zinc and function as an E3 ligase.

## Discussion

In this report, we describe two families with two related BP‐associated AFF cases in each. This is the first study using WES to describe genetic findings from familial BP‐associated AFF cases of Asian ethnicity. Using a candidate gene approach, variants of interest were identified in the *PLOD2* and *TMEM25* genes. By inheritance model–based approach, 132 variants were identified with a dominant model and one rare homozygous variant and three compound heterozygous variant pairs were identified with a recessive model, which have not been closely linked to AFF cases in the current literature. We have provided a list of potential rare variants that may be useful for future genetic studies conducted in AFF cohorts.

Using the candidate gene approach, comprising a list of genes linked to AFF in the literature, we identified two heterozygous variants of interest in *PLOD2* and *TMEM25*. A rare heterozygous variant (rs776654051; p.Thr419Ser) in *PLOD2* was identified in the single patient 1‐b of the mother‐daughter AFF family. DNA was not available from the mother (1‐a) to confirm whether this variant is shared. Homozygous mutations in *PLOD2* cause Bruck syndrome 2 (MIM609220),^(^ ^31^ ^)^ a rare form of osteogenesis imperfecta, including clinical features of short stature, bone abnormalities, osteopenia, and bone fragility. *PLOD2* codes for telopeptide lysyl hydroxylase, a protein important for hydroxylysine aldehyde crosslinking of bone collagen.^(^ ^32^ ^)^ Although BPs are associated with increased non‐enzymatic cross‐linking, which decreases bone strength,^(^ ^33^ ^)^ the added effects of reduced hydroxylysine cross‐linking might contribute to collagen deformation and thus to AFF.

BP‐associated AFFs have been reported in individuals with osteogenesis imperfecta; however, the specific gene implicated has not always been provided. A direct link between the *PLOD2* gene and AFF has not been previously reported; however, a tibial diaphyseal fracture with radiological features similar to AFF has been described in a *PLOD2*‐related osteogenesis imperfecta case due to a homozygous variant p.Trp588Cys.^(^ ^31^ ^)^

Another rare variant was identified in the gene *TMEM25* (rs782188288; p.Ala49Thr) and shared by both sisters (2‐a, 2‐b), but not by case 1‐b. Roca‐Ayats and colleagues^(^ ^17^ ^)^ also describe a variant in this gene, which was shared by their three studied sisters (a deletion of one amino acid: p.V239del not reported in gnomAD). *TMEM25* encodes Transmembrane Protein 25 (TMEM25). TMEM25 was identified as a member of the immunoglobulin superfamily,^(^ ^34^ ^)^ considered a tumor suppressor gene,^(^ ^35^ ^)^ and demonstrated to regulate neuronal excitability by modulating Nr2b‐mediated currents in neurons.^(^ ^36^ ^)^ A link between this gene and AFF, or a bone phenotype, has not been reported in the literature and different (clinical) databases, but it is interesting that both familial AFF studies report a rare variant in the same gene.

Except these two variants, no other variants were identified in the candidate gene analysis, including the analysis of the *GGPS1* and *CYP1A1* genes, which were implicated to be associated with BP‐associated AFF by a genetic study of three sisters affected with BP‐associated AFFs and subsequent functional studies.^(^ ^17^, ^37^ ^)^

The unbiased variant filtering approach assuming a dominant inheritance model on the biallelic variants where both sisters (2‐a and 2‐b) share the same heterozygous variants resulted in 132 variants with a frequency <0.005 in both the overall population and the East Asian subpopulation in public databases. Potential causal variant selection in WES data in only two samples leaves many variants to select from; hence, we prioritized variants predicted to be most damaging (truncating, or CADD >30) as potential candidates, because these often disrupt the function of the protein and are known to cause Mendelian disorders. Seven variants that fall into these two groups are in the *EPHA10*, *SORCS1*, *GSX2*, *PARP2*, *C19orf60*, *APH1B*, and *PHYHD1* genes. In addition, although none of the variants was also shared by 1‐b in the first family, one or more different variant(s) was present in 1‐b in *TTN*, *FAN1*, *ANKS3*, and *IL2RG*. However, none of these genes have a documented function related to bone. Moreover, the *TTN* gene was also identified by Garcia‐Giralt and colleagues,^(^ ^26^ ^)^ reporting eight AFF patients carrying possibly damaging variants in this gene. This is likely a chance finding because the *TTN* gene is one of the largest genes in the genome, which encodes the largest known protein. The other gene, *FSIP2*, with a variant carried by both 2‐a and 2‐b and also presented by Garcia‐Giralt and colleagues,^(^ ^26^ ^)^ encodes a protein that is specific to spermatogenic cells.^(^ ^38^ ^)^

As an example of how one could further prioritize from a long list of 132 variants, we compared these variants with specific pathways within the KEGG database. The presence of TRAF2 and TRAF6 in the osteoclast differentiation pathway revealed a potential candidate (GLy35Arg missense, NM_004295.4) present in 2‐a and 2‐b in the *TRAF4* gene, which encodes a protein in the same family. TRAF proteins belong to a family of cytoplasmic adaptors that interact directly or indirectly with TNF receptors to mediate a signaling cascade and activation of nuclear factor‐κB (NF‐κB) and c‐Jun N‐terminal kinase (JNK) pathways. TRAF4 is an adaptor protein and signal transducer that links members of the TNF receptor family to different signaling pathways, which is also required for normal skeletal development. Importantly, TRAF4‐deficient mice have rib, sternum, and spinal column malformations including scoliosis and kyphosis.^(^ ^39^ ^)^ TRAF4 can regulate the osteogenic process of mesenchymal stem cells by acting as an E3 ubiquitin ligase to degrade Smurf2, a ligase that interacts with, and degrades, essential osteogenesis‐related molecules including Smad1 and Runx.^(^ ^40^ ^)^ The RING domain mediates a crucial step in the ubiquitination pathway by simultaneously binding ubiquitination enzymes and their substrates and hence functioning as an E3 ligase. TRAF4 RING deletion mutants are reported to have lost the ability to degrade Smurf2 or to ubiquitinate Smurf2.^(^ ^40^ ^)^ Thus, the TRAF4 Gly35Arg variant may have impaired E3 ubiquitin ligase activity affecting the degradation of Smurf2 leading to dysregulation of the osteogenesis factors Smad1 and Runx2. However, apart from being a useful example, we have no evidence that this gene is implicated in AFF because the two sisters shared 50% of their genetic information. It could equally be related to the indication for BP therapy or irrelevant to both. The finding should be replicated in other families or isolated cases with reference to proper controls before confirming with a functional study.

The recessive model analysis on the biallelic variants resulted in a missense variant in the *MANF* gene. The mesencephalic astrocyte derived neurotrophic factor (MANF) protein is located in the endoplasmic reticulum (ER) and potentially modulates ER stress responses. It has been shown that MANF is important for cartilage development and hence long‐bone growth, but it has not been documented to influence bone quality.^(^ ^41^ ^)^ Compound heterozygous variants shared by 2‐a and 2‐b were identified in *SYNPO2L*, *TTN*, and *GSX2*. SYNPO2L is an acting binding protein.^(^ ^42^ ^)^ Loss‐of‐function mutations in *SYNPO2L* have been associated with atrial fibrillation,^(^ ^43^ ^)^ but a relation to bone has not been documented. *GSX2* has been implicated to be involved in the Notch signaling pathway.^(^ ^44^ ^)^ Although the Notch signaling pathway plays an important role in bone remodeling,^(^ ^45^ ^)^ *GSK2* has not been linked directly to bone homeostasis.

We acknowledge that there are several limitations to our analysis, such as the small sample size, lack of genetic data from appropriate control groups, and the inability to obtain genetic data from 1‐a. The variants of interest described in this report remain speculative and lack functional data, but we provide a comprehensive list of rare variants in two Asian families with AFFs and describe methods of filtering/analysis including a candidate gene list specific for AFF cases that may be useful for guiding future studies.

## Conclusion

BP use in osteoporosis leading to AFF is rare but could be conferred by genetic susceptibility. A gene implicated in AFF has not been consistently identified in the numerous published studies and case reports on AFF cases/cohorts. The rarity of AFFs may hinder genetic studies, because large cohorts with available genetic data are needed in order to conduct adequately powered analyses. Therefore, studying small families with AFF is essential and publishing rare variant lists and methods of analysis in AFF family clusters may aid future studies in prioritizing genes in AFF cohorts. Although our findings are inconclusive, the aggregation of AFFs in families lends support to the hypothesis that genetic factors contribute to AFF risk and provides motivation for future genetic studies in larger cohorts of familial and unrelated AFF cases, taking into account potential differences related to ethnic background.

## Author Contributions

**Wei Zhou:** Data curation; formal analysis; methodology; visualization; writing – original draft; writing – review and editing. **Hanh H Nguyen:** Data curation; formal analysis; project administration; resources; writing – original draft; writing – review and editing. **Denise M van de Laarschot:** Methodology; project administration; writing – review and editing. **Tet Sen Howe:** Resources; writing – review and editing. **Joyce S.B Koh:** Resources; writing – review and editing. **Frances Milat:** Resources; writing – review and editing. **Jeroen G.J. van Rooij:** Supervision; writing – review and editing. **Joost A.M. Verlouw:** Data curation; software; writing – review and editing. **Bram C.J. van der Eerden:** Writing – review and editing. **Mark Stevenson:** Formal analysis; methodology; visualization; writing – review and editing. **Rajesh V Thakker:** Methodology; supervision; writing – review and editing. **M. Carola Zillikens:** Conceptualization; funding acquisition; project administration; supervision; writing – review and editing. **Peter R Ebeling:** Conceptualization; funding acquisition; project administration; resources; supervision; writing – review and editing.

## Conflicts of Interest

PRE and MCZ received research funding from NHMRC and PRE from Amgen for this study.

### Peer Review

The peer review history for this article is available at https://publons.com/publon/10.1002/jbm4.10659.

## Supporting information


**Fig. S1** Sanger sequencing validation for the different identified variants. Samples 2‐a and 2‐b show a heterozygous variant for rs782188288 in *TMEM25* (column4), sample 1‐b shows a heterozygous variant for rs776654051 in *PLOD2* (column3). The other samples are reference sequence. Green indicates A, Black indicates G, Red indicates T, Blue indicates C.

**Table S1** Variants heterozygously shared by 2‐a and 2‐b, irrespective of 1‐b
**Table S2** Different variants in the same gene shared by 1‐b, 2‐a and 2‐b
**Table S3** Homozygous variants or compound heterozygous variants shared by 2‐a and 2‐b, irrespective of 1‐b
**Table S4** Multiallelic variants homozygously/heterozygously shared by 2‐a and 2‐b, irrespective of 1‐b
