# Supplementary material for: Whole Exome Sequencing in Two Southeast Asian Families With Atypical Femur Fractures
Source: JBMR Plus. 2022 Jul 3;6(8):e10659. doi: 10.1002/jbm4.10659 (PMC9382867; doi:10.1002/jbm4.10659)
Supplement: Supplementary file 1 — Fig. S1 Sanger sequencing validation for the different identified variants. Samples 2‐a and 2‐b show a heterozygous variant for rs782188288 in TMEM25 (column4), sample 1‐b shows a heterozygous variant for rs776654051 in PLOD2 (column3). The other samples are reference sequence. Green indicates A, Black indicates G, Red indicates T, Blue indicates C. [file JBM4-6-e10659-s001.docx]

**Supplementary Figure 1:** Sanger sequencing validation for the different identified variants.

samples 2-a and 2-b show a heterozygous variant for rs782188288 in *TMEM25* (column4), sample 1-b shows a heterozygous variant for rs776654051 in *PLOD2* (column3). The other samples are reference sequence. Green indicates A, Black indicates G, Red indicates T, Blue indicates C.


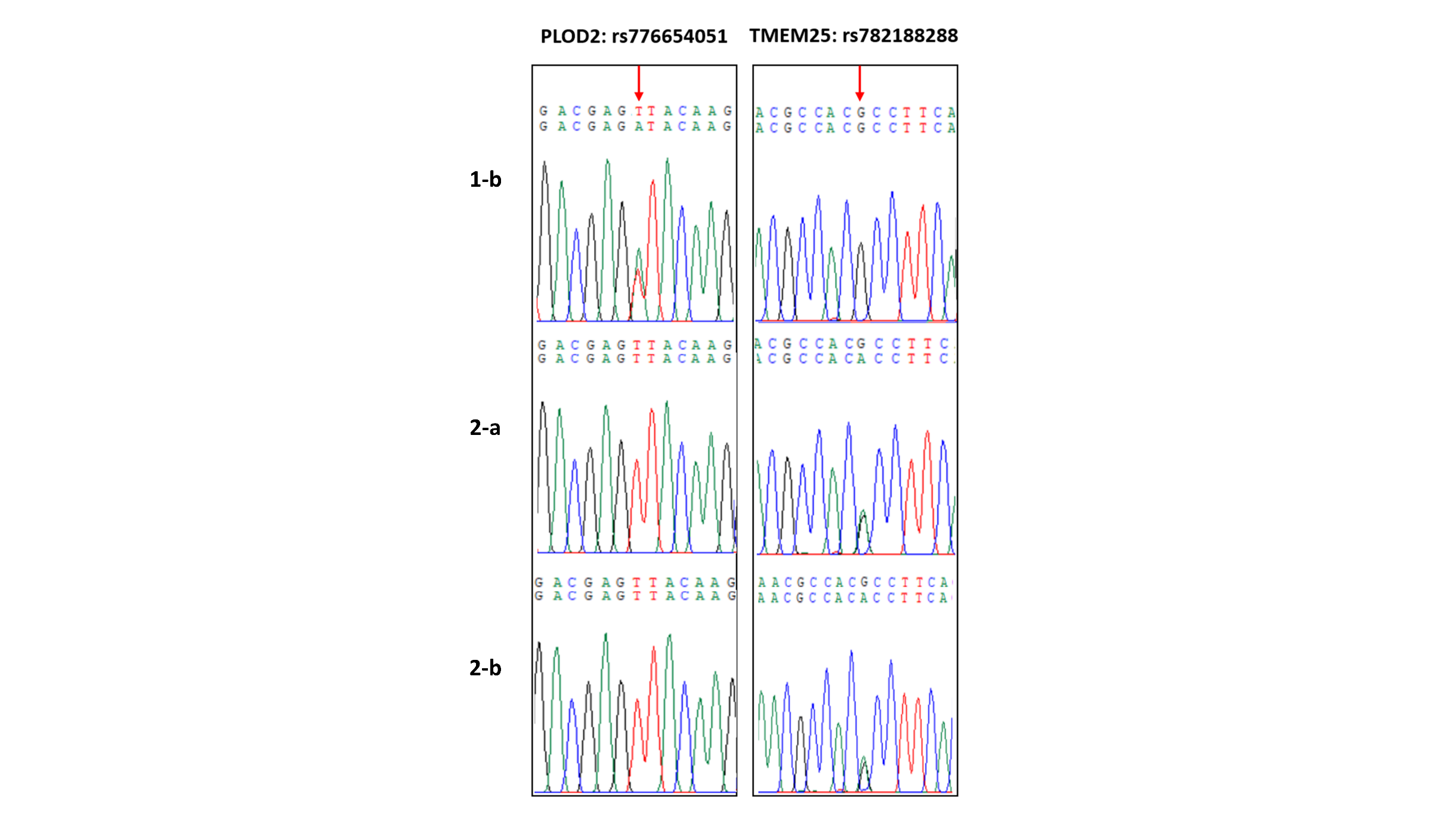


**Primers used for variants verified by Sanger Sequencing**

| Gene | Variant | Forward primer (5’ to 3’) | Reverse primer (5’ to 3’) |
| --- | --- | --- | --- |
| *PLOD2* | rs776654051 | GCAATGAGCTTGTTCCTTTGA | CTACAGGTTTGTTGAATGAGC |
| *TMEM25* | rs782188288 | TCTCTCCCCTGTCTGCACTTC | TGCACATTAAGGATGACAGAG |
